# Description of a new species of *Coelosis* Hope from Guajira Peninsula, northern Colombia (Coleoptera, Scarabaeidae, Dynastinae, Oryctini)

**DOI:** 10.3897/zookeys.738.22273

**Published:** 2018-02-19

**Authors:** Jhon César Neita-Moreno, Jesús Orozco, Claudia Alejandra Medina-Uribe

**Affiliations:** 1 Colecciones Biológicas-Entomología, Subdirección de Investigaciones, Instituto de Investigaciones de Recursos Biológicos Alexander von Humboldt, Claustro de San Agustín, Villa de Leyva, Boyacá, Colombia; 2 Insect Collection, Agricultural Science and Production Department, Zamorano University, Zamorano, Honduras; 3 Instituto de Investigaciones de Recursos Biológicos Alexander von Humboldt, Claustro de San Agustín, Villa de Leyva, Boyacá, Colombia

**Keywords:** Distribution, Macuira Mountains, Scarabaeoidea, Taxonomy, Distribución, Serranía de la Macuira, Scarabaeoidea, Taxonomía

## Abstract

A new species of *Coelosis* is described from the Macuira Mountains, Guajira Peninsula, northern Colombia. A character comparison between this new and other previously known Colombian species in the genus is presented. A key for the identification and distributional map for Colombian species of *Coelosis* is provided, as well as a key for the genera included in the tribe Oryctini in Colombia.

## Abstract (es)

Una nueva especie de *Coelosis* es descrita de la Serranía de la Macuira en la Peninsula de la Guajira

en el extremo norte de Colombia. Se ilustran los caracteres diagnósticos y se compara la nueva especie con las especies previamente reportadas para Colombia. Se provee una clave para la identificación y un mapa de distribución de las especies de *Coelosis* en el país al igual que una clave para los géneros de la tribu Oryctini en Colombia.

## Introduction

The genus *Coelosis* is a Neotropical dynastine group of mainly nocturnal forest beetles that can be collected at lights (Endrödi 1985, Iannuzzi and Marinoni 1996, Ratcliffe 2003). The larvae of *Coelosis biloba* (L.) is the only one described in the genus and is known to be associated with the nests of the leaf-cutter ant *Atta cephalotes* (L.) (Hymenoptera: Formicidae) (Pardo-Locarno et al. 2006).

The purpose of this work is to describe a new species of *Coelosis* from a tropical dry forest in the protected area of Macuira, Guajira Pensinsula, to provide new biological data and distribution records for other species *Coelosis*, and to provide diagnostic characters and illustrations for all three Colombian species in the genus *Coelosis* as well as a key to the genera of Oryctini from Colombia.

## Materials and methods

Internal and external morphological characters were studied using a dissecting microscope (6.5–40.0×). For measurements, an ocular micrometer was used. Internal sclerotized structures were dissected after relaxing the specimen in hot (75 °C) water. Heavily sclerotized parts were soaked in a 15 % solution of potassium hydroxide and neutralized in a 15 % solution of acetic acid. Genitalia were card-mounted or placed in a glycerin‒filled vial beneath the specimen.

Specimens were characterized using body length, puncture density, setation, and color as described in Orozco (2012).

### Material examined

138 specimens were reviewed and label data were obtained from the following Colombian collections except where noted otherwise (curators in brackets):

**ANDES** Colección de Entomología, Universidad de Los Andes, Bogotá D.C. (Oscar Mahecha).

**ICN‒MHN** Colección de Zoología, Instituto de Ciencias Naturales, Universidad Nacional de Colombia, Bogotá D.C. (Germán Amat-García).

**IAvH** Colecciones Biológicas, Instituto de Investigaciones de Recursos Biológicos Alexander von Humboldt, Villa de Leyva, Boyacá.

**LGA** Museo de Historia Natural “Luis Gonzalo Andrade”. Colección Entomológica. Universidad Pedagógica y Tecnológica de Colombia, Tunja, Boyacá (Fredy Molano).

**MEFLG** Museo Entomológico Francisco Luis Gallego, Universidad Nacional de Colombia, sede Medellín, Antioquia (Jhon Alveiro Quiroz).

**MEUC** Museo de Entomología, Universidad de Cundinamarca, Fusagasugá, Cundinamarca.

**MLP** Museo de La Plata, Colección de Entomología, La Plata, Buenos Aires, Argentina (Nhora Cabrera and Analía Lanteri).

**MPUJ** Museo Javeriano de Historia Natural ”Lorenzo Uribe”, Pontificia Universidad Javeriana, Bogotá, D.C. (Dimitri Forero).

**MUA** Colección de Ciencias Naturales, Universidad de Antioquia, Medellín, Antioquia (Marta Wolf).

**MUSENUEV** Museo Entomológico, Universidad del Valle, Cali, Valle del Cauca (James Montoya).

**UNAB** Museo Entomológico, Facultad de Agronomía, Universidad Nacional de Colombia, Bogotá, D.C. (Francisco Serna y Erika Vergara).

Additional locality information was obtained from Sanabria-García et al. (2012). ArcMap 10.0 (ESRI 2011) was used to build the distributional map.

## Results

### Key to the genera of Oryctini from Colombia

**Table:** 

1	Labium subtriangular or with bulging lateral margins (Fig. 2E, F, G). Mandibles with 1‒2 teeth on external margin (Fig. 2H–J). Second segment of maxillary palp longer than first and third segments (Fig. 2A–D)	**2**
–	Labium subrectangular, lateral margins subparallel (Fig. 4C, F, I). Mandibles with three teeth on external margin (Fig. 4A, B, D, E, G, H). Second segment of maxillary palp similar in length to first and third segment (Fig. 5A, B, C)	***Coelosis* Hope**
2	Maxilla with 5‒6 teeth (Fig. 2C, D)	**3**
–	Maxilla with 1‒3 teeth (Fig. 2A, B)	**5**
3	Male with one pronotal horn and one large cephalic horn or tubercle (Fig. 1F). Female without fovea on pronotum	***Podischnus* Burmeister**
–	Male with three pronotal horns and/or tubercles and lacking large cephalic horn. Female with fovea on pronotum	**4**
4	Elytra smooth (Fig. 1E)	***Strategus* Kirby**
–	Elytra with rows of deep punctures (Fig. 1B)	***Gibboryctes* Endrödi**
5	Protibia tridentate (Fig. 1D). Apex of labium pointed, paraglossa undeveloped (Fig. 2E)	***Megaceras* Hope**
–	Protibia quadridentate (Fig. 1A, B, E, F). Apex of labium blunt, paraglossa developed (Fig. 2F, G)	**6**
6	Mandibular teeth widely separated at base (Fig. 2H). Males and females with cephalic horns (Fig. 1A)	***Enema* Hope**
–	Mandibular teeth contiguous or fused at base (Fig. 2I). Cephalic horns present only in males	***Heterogomphus* Burmeister**

**Figure 1.**
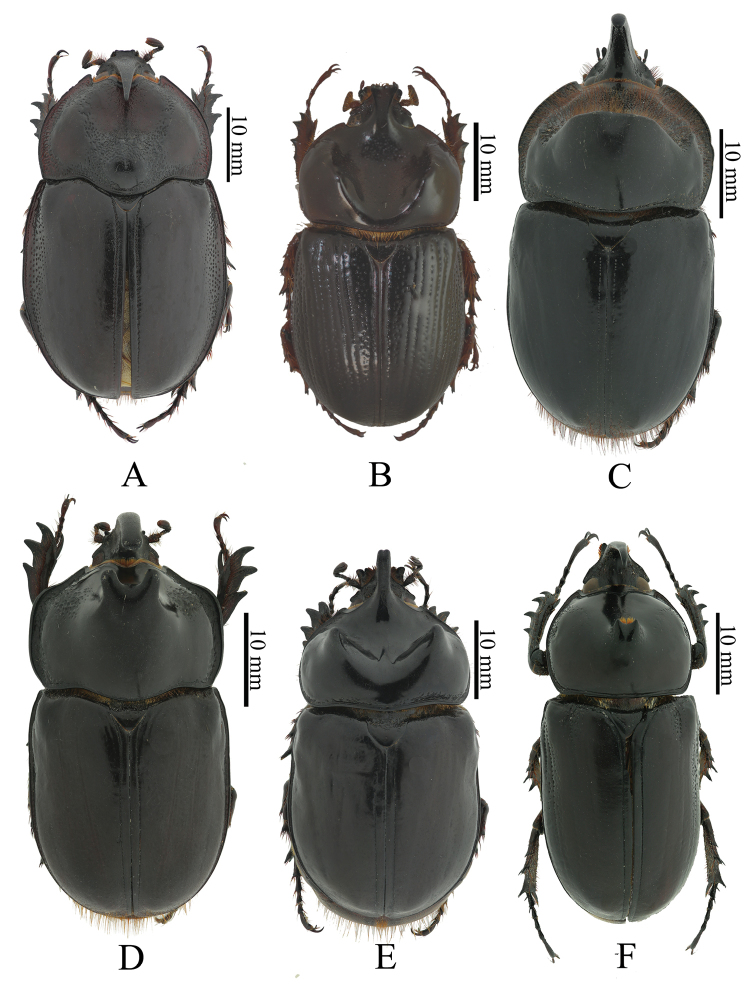
Colombian Oryctini (dorsal habitus). **A** *Enema pan* (Fabricius) **B** *Gibboryctes waldenfelsi* (Endrödi) **C** *Heterogomphus chevrolati* Burmeister **D** *Megaceras porioni* Dechambre **E** *Strategus fascinus* Burmeister **F** *Podischnus agenor* (Olivier).

**Figure 2.**
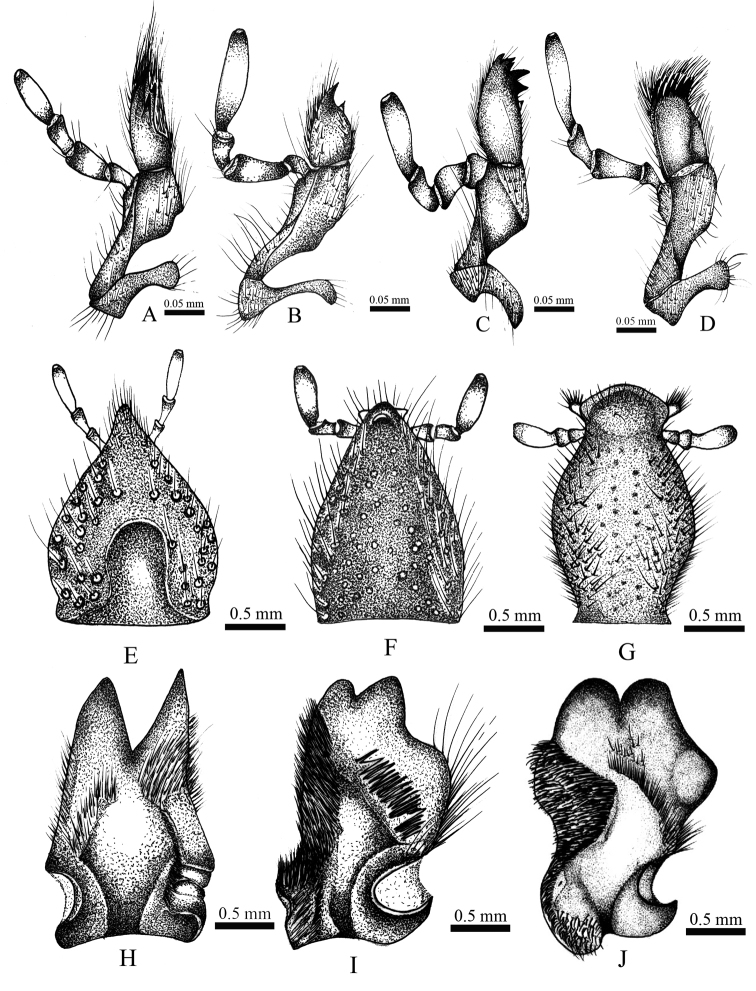
**A, H** *Enema pan* **B, F, I** *Heterogomphus chevrolati* **C** *Gibboryctes waldenfelsi* **D** *Podischnus agenor* **E** *Megaceras morpheus* Burmeister **G, J** *Strategus fascinus* **A–D** maxilla (dorsal view) **E–G** labium (ventral view) **H–J** mandibles (dorsal view).

### Key to adults of the species of *Coelosis* from Colombia

**Table:** 

1	Maxilla without lateral projection (Fig. 5B)	***Coelosis biloba* (L.)**
–	Maxilla with lateral projection (Fig. 5A, C)	**2**
2	Galea with two teeth (Fig. 5A). Prohypomeron and metasternum with long setae (Fig. 6F)	***Coelosis bicornis* (Leske)**
–	Galea without teeth (Fig. 5C). Prohypomeron and metasternum with short setae (Fig. 6G)	***Coelosis wayuorum* sp. n.**

#### Coelosis wayuorum sp. n.

Taxon classificationAnimaliaColeopteraDynastidae

http://zoobank.org/863A21DA-0365-4404-B1BF-C125EE00B609

**Figure 3.**
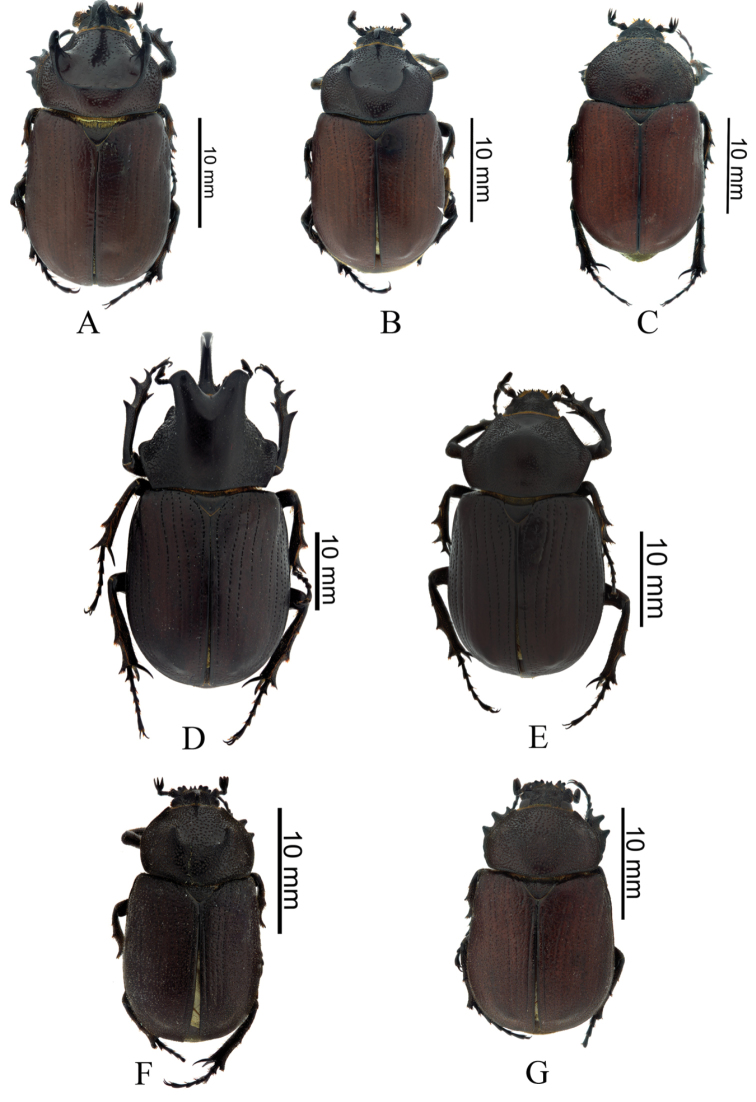
*Coelosis* species from Colombia (dorsal habitus): **A** (male major) **B** (female) **C** (male minor): *C. bicornis* **D** (male major): *C. biloba* (L.) **E** (female): *C. biloba* **F** Holotype (male): *C. wayuorum* sp. n. **G** Allotype (female): *C. wayuorum* sp. n.

**Figure 4.**
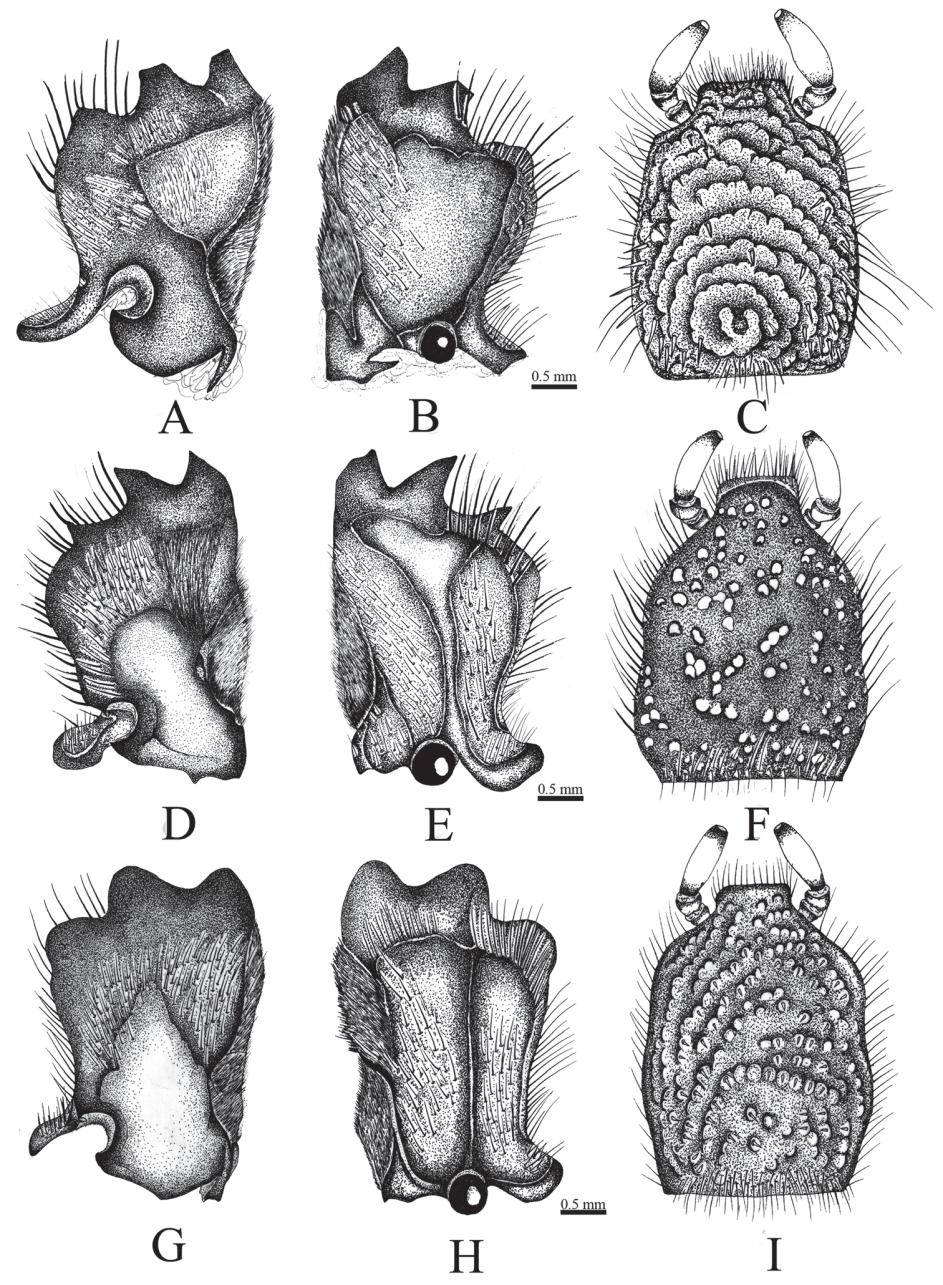
**A, B, C** *Coelosis bicornis* **D, E, F** *C. biloba* **G, H, I** *C. wayuorum* sp. n. **A, D, G** mandibles (dorsal view) **B, E, H** mandibles (ventral view) **C, F, I** labium (ventral view).

**Figure 5.**
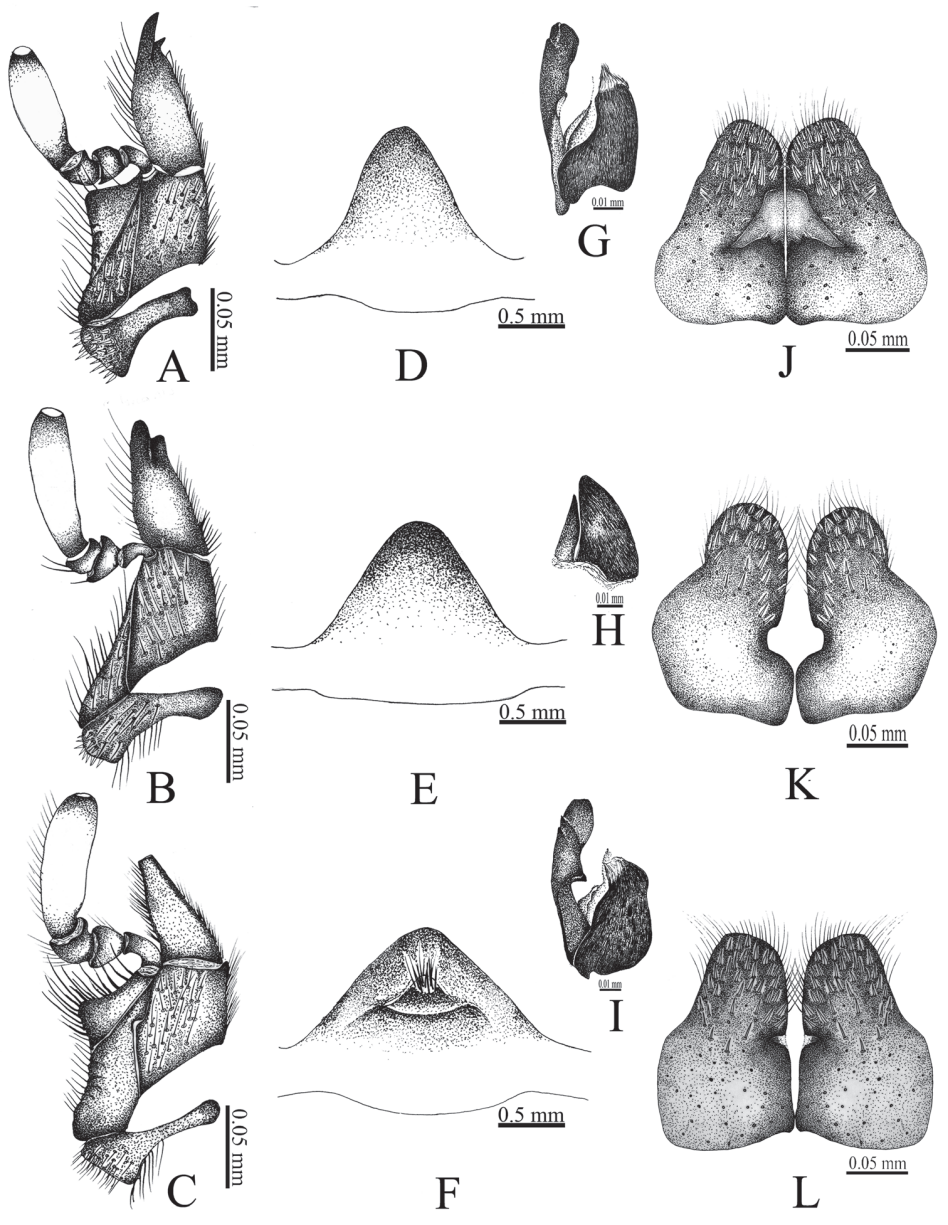
**A, D, G, J** *Coelosis bicornis* **B, E, H, K** *C. biloba* **C, F, I, L** *C. wayuorum* sp. n. **A–C** maxilla (ventral view) **D–F** prosternal process (antero-posterior view) **G–I** internal sac (copulatory lamellae) **J–L** genital plate (female).

**Figure 6.**
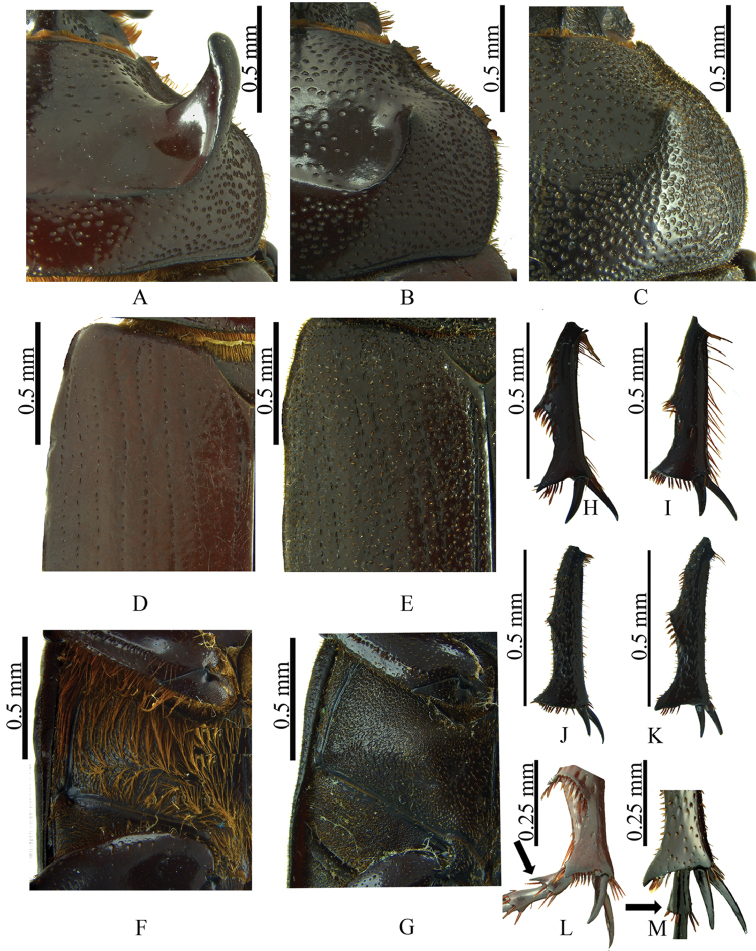
**A, B, D, F, H, I, L** *Coelosis bicornis* **C, E, G, J, K, M** *Coelosis wayuorum* sp. n. **A–C** pronotum (dorsal view) **D, E** elytra **F, G** metasternum **H, J** mesotibia (dorsal view) **I, K** metatibia (dorsal view) **L, M** first metatarsomere (dorsal view).

**Figure 7.**
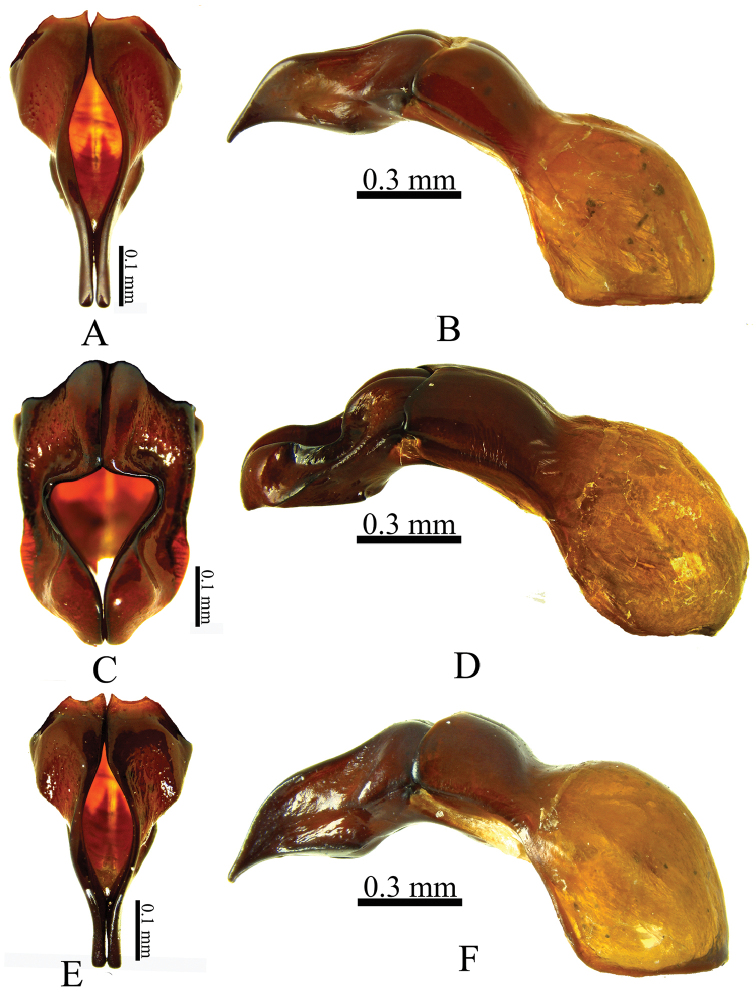
**A, B** *Coelosis bicornis* **C, D** *C. biloba* **E, F** *C. wayuorum* sp. n. **A, C, E** parameres (frontral view) **B, D, F** phallobase (lateral view).

**Figure 8.**
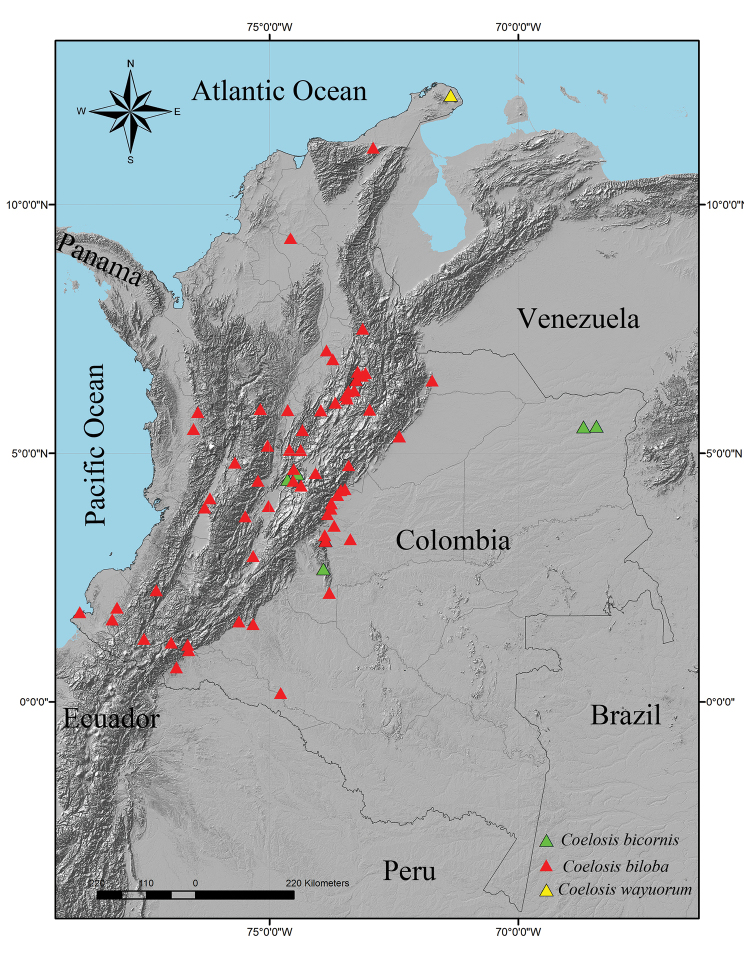
Distribution of *Coelosis* in Colombia.

Figs 3F–G ; 4G–I ; 5C, F, I, L ; 6C, E, G, J, K, M ; 7E–F ; 8

##### Type material

**(3)**. Holotype labeled “Colombia, La Guajira, Uribia, PNN La/Macuira Corregimiento Nazareth/Kajashiwoü, 12°11'37.9”N; 71°21'30.1”/W. WGS84, 70 m. Manual.19.ix.2014/C. Medina” [IAvH-E-195379]. Allotype [IAvH-E-195380] and one female paratype [IAvH-E-195381] with the same label data. Types deposited at the Instituto Alexander von Humboldt (IAvH) Villa de Leyva, Boyacá, Colombia.

##### Diagnosis.

*Coelosis wayuorum* sp. n. can be separated from the other Colombian *Coelosis* by the following characters: maxilla with lateral sclerite pronounced (Fig. 5C) [similar to *C. bicornis*, but not as in *C. biloba*]; galea without teeth (Fig. 5C) [two teeth in both *C. bicornis* and *C. biloba*]; ventral surface of the mandibles with keels 1 and 2 contiguous (Fig. 4H) [widely separate in *C. bicornis* (Fig. 4B), slightly separate in *C. biloba* (Figs 4E)]; pronotum and elytra strongly punctate, punctures with short, spine-like setae (Figs 6C, E) [spine-like setae absent in both *C. bicornis* and *C. biloba* (Fig. 6A, B, D)]; prohypomeron with spine-like setae [slender and long in *C. bicornis*, slender and short in *C. biloba*]; mesosternum convex as in *C. biloba* [concave in *C. bicornis*]; metasternum covered with short, spine-like setae (Fig. 6G) [setae long and slender in both *C. bicornis* and *C. biloba* (Fig. 6F)]; and meso- and metatibiae densely punctate (Fig. 6J, K) [scarcely punctate in both *C. bicornis* and *C. biloba* (Fig. 6H, I)]. The internal sac is different among the species: in *C. biloba*, the accessorial lamella is short and simple (Fig. 5H), while in *C. bicornis* and *C. wayuorum* sp. n. the lamella is long and complex, although with differences between these two species (Fig. 5G, I).

##### Description.

Holotype male (Fig. 3F). Body length 20.2 mm; width 11.00 mm. Color dark reddish brown. *Head*: Frontoclypeal region with small horn, surface rugopunctate, punctures setose, setae spine-like (Fig. 3F). Clypeus strongly rugopunctate, slightly emarginate, apex broad with two reflexed teeth. Mandibles with three conical teeth, subapical notch slightly deep (Fig. 4G, H); labium rugopunctate, with sparse, short, spine-like setae, paraglossa undeveloped, narrow, apex truncate (Fig. 4I); maxilla with lateral projection, galea without teeth (Fig. 5C). *Pronotum*: Surface sparsely punctate; punctures moderately large, umbilicate, setose; setae spine-like (Fig. 6C). Disc with two small, widely separated horns, wide fovea between horns (Figs 3F, 6C). *Elytra*: Surface with 10 distinct pairs of striae composed of ocellate punctures bearing short, spine-like setae, micropunctures densely intermixed between striae (Fig. 6E). *Pygidium*: Surface sparsely punctate, punctures small to moderate in size, weakly ocellate, with short spine-like setae, becoming denser at basal angles. Surface regularly convex in lateral view. *Legs*: Protibia tridentate, basal tooth reduced. Meso- and metatibiae with one medial transverse carina, each with short, spine-like setae and one small inner tooth (Fig. 6J, K). First metatarsomere apically expanded, apex subtruncate with acute outer projection (Fig. 6M). *Venter*: Prosternal process subtriangular, thick; apex short, parabolic, with process at middle, this process with short, stout setae (Fig. 5F). Mesosternum densely punctate, setose, slightly convex at middle. Metasternum densely punctate; punctures ocellate, minutely setose; setae spine‒like; lateral edge rugopunctate with short, spine-like setae. Abdominal ventrite VIII depressed at middle. *Genitalia*: Parameres as in Fig. 7E, F, internal sac as in Fig. 5I.

##### Allotype

(Fig. 3G). Female. Similar to holotype except for the following: body length 22.4 mm; width 12.0 mm, frons and disc of pronotum smooth, without fovea or horns, pygidium slightly concave in lateral view, and genital plate as Fig. 5L.

**Paratype (1).** Similar to allotype except for the following: body length 25.5 mm; width 13.0 mm.

##### Etymology.

The specific epithet *wayuorum* refers to the Wayuu indigenous group inhabiting the Guajira Peninsula.

##### Distribution.

*Coelosis wayuorum* sp. n. is known only from one locality in Macuira National Park, Colombia (Fig. 8).

##### Temporal distribution.

Three specimens collected in September 2014, during the dry season.

##### Life history.

The type material was collected at night with lights during the dry season.

### The following are new biological data and distribution records for *Coelosis* species in Colombia

***Coelosis bicornis***. **Vichada**, Municipio de Puerto Carreño, Vereda La Esmeralda. El Tomo. 5.554252 Lat. -68.467042 Long. 81 msnm. Trampa de Luz Negra. 31 de marzo a 9 de abril de 2017. J. C. Neita, A. Lopera & J. Cárdenas [IAvH-E-198889 (1♂) and IAvH-E-198890 (1♀)].

Adults of *C. bicornis* have been captured in nests of *Atta laevigata* (Smith, 1858) (Hymenoptera: Formicidae) in the Orinoco basin.

***Coelosis biloba.* Antioquia**, Carmen de Viboral, El Porvenir, Finca La Samaria, bosque de restauración, 05°53'15.2"N; 075°11'11.8"W. 1000‒1100. WGS84 2016 11 30. A. Lopera & J. Cárdenas [IAvH-E-198891 (1♂), IAvH-E-198892 (1♀), IAvH-E-198893 (1♀)]. **Chocó**, Quibdó, Corregimiento Tutunendó. Finca cerca al pueblo. 5°44,58'N; 76° 32,043'W. 68 m snm. Trampa de Luz Negra. Abril de 2000. J. C. Neita Leg. [IAvH-E-198893 (1♂), IAvH-E-198894 (1♂), IAvH-E-198895 (1♂), IAvH-E-198896 (1♂), IAvH-E-198897 (1♀), IAvH-E-198898 (1♀), IAvH-E-198899 (1♀)]. **Chocó**, Quibdó, Corregimiento de Pacurita. Pueblo. 5°41'N; 76°40'W. 43 m snm. Trampa de Luz Negra. Febrero de 2001. J. C. Neita Leg. [IAvH-E-198900 (1♂), IAvH-E-198901(1♂), IAvH-E-198902 (1♀)]. **Chocó**, Quibdó. Corregimiento de Tutunendó, Estación Biológica Ambiental IIAP. 5°40'23.04”N; 76°33'42.38”W. 82 m alt. Ene. 2010. J. C. Neita Leg. [IAvH-E-198903 (1♂), IAvH-E-198904 (1♂), IAvH-E-198905 (1♂), IAvH-E-198906 (1♂), IAvH-E-198907 (1♂), IAvH-E-198908 (1♀), IAvH-E-198909 (1♀), IAvH-E-198910 (1♀), IAvH-E-198911 (1♀),].

## Supplementary Material

XML Treatment for Coelosis wayuorum
